# Supplementary material for: LazyNet: Interpretable ODE Modeling of Sparse CRISPR Single-Cell Screens Reveals New Biological Insights
Source: Biology (Basel). 2025 Dec 29;15(1):62. doi: 10.3390/biology15010062 (PMC12785065; doi:10.3390/biology15010062)
Supplement: Supplementary file 1 [file biology-15-00062-s001.zip › Supplement_S4.pdf]

Supplement S4 — Mathematical Details of LazyNet and Elasticity-Based Network Inference

S4.1 Notation and data model

Let $x \in \mathbb{R}_{>0}^p$ denote gene expression (counts or normalized abundances), $u \in \{0,1\}^p$ an intervention indicator (one-hot or multi-hot guide vector), and $\varepsilon>0$ a small pseudocount ensuring positivity. We work in the log domain so that fold-changes become additive. All logarithms are elementwise.

$$z = \log(x + \varepsilon) \in \mathbb{R}^p \quad \text{(S4.1)}$$

For two-snapshot experiments, each cell provides a triple (z_{pre}, u, z_{post}); “pre”/“post” refer to the baseline and post-perturbation states after library-size normalization and pseudocount shift.

S4.2 One-step log–exp residual update (Euler discretization)

LazyNet instantiates a single explicit ODE step in the log domain via a residual map $g\theta(\cdot)$:

$$z_{post} = z_{pre} + g\theta(z_{pre}, u) \quad \text{(S4.2)}$$

In expression space, the residual is multiplicative due to the log–exp change of variables:

$$x_{post} = (x_{pre} + \varepsilon) \odot \exp(\, g\theta(\, \log(x_{pre} + \varepsilon), u \,)\,) - \varepsilon \quad \text{(S4.3)}$$

Equation (S4.2) is the Euler step of $\dot{x}$ in log space with an unknown step size Δt. Writing $f\theta$ for the continuous-time field,

$$dz/dt = f\theta(z, u), \quad g\theta(z, u) = \Delta t \cdot f\theta(z, u) \quad \text{(S4.4)}$$

Hence only the product $\Delta t \cdot f\theta$ is identifiable from two snapshots; absolute Δt is not determined.

S4.3 Exact representation of multiplicative synergy via the log–linear–exp map

If the residual is affine in (z, u), $g\theta(z, u) = A z + B u + b$, then a linear form in z produces exact power-law interactions in x (elementwise):

$$x_{post,i} = (x_{pre,i} + \varepsilon) \cdot \Pi_j \, (x_{pre,j} + \varepsilon)^{A_{ij}} \cdot \exp(\, \Sigma_k B_{ik} u_k + b_i \,) - \varepsilon \quad \text{(S4.5)}$$

Thus pairwise and higher-order multiplicative effects appear as explicit model components. With smooth hidden layers, $g\theta$ represents mixtures of power laws (log-sum-exp features), preserving multiplicative semantics.

S4.4 Training objective (robust regression)

Main runs use the Huber loss with $\delta = 0.1$ on the modeling gene panel $\mathcal{G}$. For residual $r = z\_post - z\_pre - g\theta(z\_pre,u)$:

$$L\_Huber(\theta) = (1/|\mathfrak{B}||\mathcal{G}|) \cdot \Sigma\_\{(\cdot)\in\mathfrak{B}\} \Sigma\_\{i\in\mathcal{G}\} [½ r\_i\text{^}2 \text{ if } |r\_i|\le\delta ;\ \delta(|r\_i| - ½\delta) \text{ otherwise}] \quad (S4.6)$$

The Huber objective soft-clips rare, extreme deviations common in perturbation screens while remaining quadratic in the bulk.

S4.5 Elasticities and Jacobians (directed, mechanism-level edges)

Define the log-elasticity matrix at a baseline b (a fixed z-vector, e.g., a cohort mean):

$$E(b,u) := \partial z\_post/\partial z\_pre\ |\_\{(z\_pre=b,u)\} = I + J(b,u), \quad \text{with} \quad J(b,u) := \partial g\theta/\partial z\ (b,u) \quad (S4.7)$$

Entry $J\_\{ij\}$ quantifies the directed local sensitivity of gene i to gene j in log space (holding u fixed). In expression space, $\partial \log x'\_i / \partial \log x\_j \approx E\_\{ij\}(b,u)$. We rank $|J\_\{ij\}|$ to summarize influence magnitudes.

S4.6 Ensemble Jacobians and subgraph extraction

Given R independently seeded checkpoints $\{\theta\text{^}(r)\}\_\{r=1..R\}$, compute $J\text{^}(r)=\partial g\theta\text{^}(r)/\partial z$ at baseline b (autodiff). The ensemble Jacobian is the elementwise average:

$$\bar{J} = (1/R) \cdot \Sigma\_\{r=1\}\text{^}R\ J\text{^}(r) \quad (S4.8)$$

Because differentiation and expectation commute under mild conditions, $\bar{J}$ estimates the expected local sensitivity. For each seed gene s, we rank both downstream ($\bar{J}\_\{\cdot s\}$) and upstream ($\bar{J}\_\{s\cdot\}$) magnitudes, retain neighbors with $|J|\ge10^{-3}$ and above the 95th or 99th percentile of the ranked list, then grow a breadth-first 32×4 consensus subgraph. For single-baseline Jacobians that yield few candidates, we omit additional FDR filtering (q=1) and report the full ranked sets.

S4.7 Identifiability and invariances

Time-step confounding: two snapshots identify $\Delta t\cdot f\theta$, not Δt itself. Scaling and shifts in x (library size, ε) enter additively in z; the residual learns on the chosen normalization. Elasticities are state-local (evaluated at b) and may vary with the baseline—an expected property of dynamic models.

S4.8 Computation and memory

Forward/backward scale with parameter count. Jacobian columns are obtained via vector-Jacobian products (autodiff) to avoid forming the full $p \times p$ matrix in memory. For storage and ranking, we keep block-sparse top-k entries per row/column and stream the remainder. This enables transcriptome-scale evaluation on CPUs.

S4.9 Evaluation linkage (module-level enrichment)

Curated databases (e.g., STRING, ARCHS4) aggregate heterogeneous and often undirected evidence, so strict edge-wise overlap is an imperfect target for directed, local elasticities. We therefore evaluate at the module level (pathways/regulons), testing whether the inferred subgraph induces coherent activity/enrichment that aligns with known biology, and we corroborate with orthogonal proteomics when available (e.g., GPX4-knockout SILAC). This yields a more stable and functionally interpretable assessment than raw edge counts.

S4.10 Two-gene toy example (exact multiplicative synergy)

Let $p=2$, $g\theta(z,u)=A z$ with $A = [[0, \alpha],[\beta, 0]]$. Then (S4.5) gives:

$$x'_1 = (x_1+\varepsilon) \cdot (x_2+\varepsilon)^\alpha - \varepsilon, \quad x'_2 = (x_2+\varepsilon) \cdot (x_1+\varepsilon)^\beta - \varepsilon \quad \text{(S4.9)}$$

Even with a linear residual in z, the update in x is multiplicative, explicitly encoding synergy between genes 1 and 2. Local elasticities at baseline b are $J = A$, so $|\alpha|$ and $|\beta|$ appear directly in the Jacobian and in the ranked neighbor lists.
