# Supplementary figures and images for: LazyNet: Interpretable ODE Modeling of Sparse CRISPR Single-Cell Screens Reveals New Biological Insights
Source: Biology (Basel). 2025 Dec 29;15(1):62. doi: 10.3390/biology15010062 (PMC12785065; doi:10.3390/biology15010062)

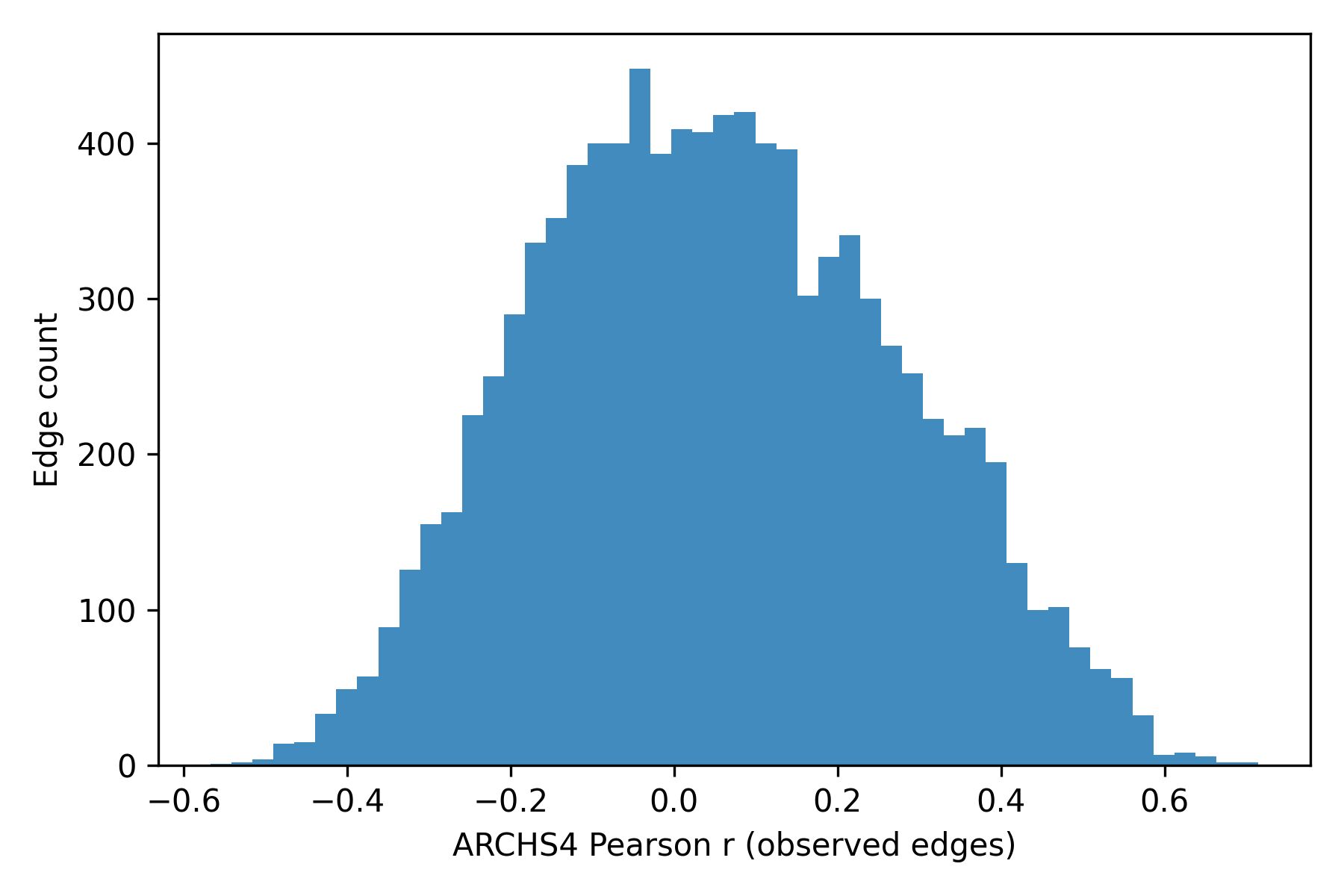

Supplement: Supplementary file 1 [file biology-15-00062-s001.zip › Supplement S2A.png]
